# Common Visual Preference for Curved Contours in Humans and Great Apes

**DOI:** 10.1371/journal.pone.0141106

**Published:** 2015-11-11

**Authors:** Enric Munar, Gerardo Gómez-Puerto, Josep Call, Marcos Nadal

**Affiliations:** 1 Human Evolution and Cognition Group, associated group to IFISC (University of the Balearic Islands–CSIC), Palma, Balearic Islands, Spain; 2 School of Psychology and Neuroscience, University of St Andrews, Saint Andrews, United Kingdom; 3 Max Planck Institute for Evolutionary Anthropology, Leipzig, Germany; 4 Department of Basic Psychological Research and Research Methods, University of Vienna, Vienna, Austria; University of Portsmouth, UNITED KINGDOM

## Abstract

Among the visual preferences that guide many everyday activities and decisions, from consumer choices to social judgment, preference for curved over sharp-angled contours is commonly thought to have played an adaptive role throughout human evolution, favoring the avoidance of potentially harmful objects. However, because nonhuman primates also exhibit preferences for certain visual qualities, it is conceivable that humans’ preference for curved contours is grounded on perceptual and cognitive mechanisms shared with extant nonhuman primate species. Here we aimed to determine whether nonhuman great apes and humans share a visual preference for curved over sharp-angled contours using a 2-alternative forced choice experimental paradigm under comparable conditions. Our results revealed that the human group and the great ape group indeed share a common preference for curved over sharp-angled contours, but that they differ in the manner and magnitude with which this preference is expressed behaviorally. These results suggest that humans’ visual preference for curved objects evolved from earlier primate species’ visual preferences, and that during this process it became stronger, but also more susceptible to the influence of higher cognitive processes and preference for other visual features.

## Introduction

Visual preference, that is to say, the choice of one item over others based on visual qualities, plays an important role in many human everyday activities and decisions [1]. For instance, visual preference guides consumer decisions [2,3], social judgment and behavior [4–6], or partner choice [7,8]. Although such preferences are sensitive to personal and contextual factors, most research has been aimed at understanding the perceptual features that drive them. These include color, combinations of colors, complexity, symmetry, as well as low-level properties, such as spatial frequency [1].

Among these attributes, one that has recently received attention is the quality of object contour; specifically, the distinction between curved and sharp-angled contours. A number of behavioral studies have shown that people prefer curved contours significantly more than sharp-angled ones [9–16]. Such a preference even seems to be present in newborns, as they preferentially fixate on curved contours, compared to sharp-angled ones [17,18].

Bar and Neta [11,12] suggested that sharp transitions in contour may convey a primitive sense of threat that triggers a primary negative response, which in turn leads to avoidance or rejection. Consistent with this hypothesis, activity in the amygdala, involved in processing information related to fear and high arousal [19], is significantly higher while viewing objects with sharp-angled contours than while viewing their curved contour counterparts [12]. Moreover, the difference in preference between curved and sharp-angled contours was more apparent with low spatial frequencies (LSFs) of the image than with high spatial frequencies (HSFs). Because the LSFs are processed faster than the HSFs [20,21], those results support the idea that the observed preference might be based on a fast extraction of information from the image at a low level of processing. In fact, several studies with adults have presented the stimuli very briefly (80–85 ms) and/or required quick responses from the participants [11,12,14], preventing top-down processing (enabled by long exposure times to the stimuli) from overriding the observed preferences. Other studies, however, have used longer presentation times, ranging from 2000 ms [13] to 3000 ms [16], or even free viewing times [10,15]. In fact, preference for curved over sharp-angled contours is not a constant that affects all our decisions about objects. There are several factors that modulate, reduce, or even eliminate such preference. Preferences for curved objects, for instance, can be partially modulated by fashion or trends, and adaptation effects are plausible candidates for triggering such changes in preference [22]. In addition, the preference for curved contours disappears when objects possess a negative emotional valence [14].

Visual preference is thought to have played an adaptive role during the evolution of the human lineage by directing our ancestors’ attention to important aspects of their natural environments [23]. From this perspective, it is believed that humans are biologically predisposed to prefer certain landscapes [24] and environmental cues [25] that contain information relative to resources and threats [26,27]. Similarly to this sort of environmental preference, it is also believed that a preference for curved contours, or a predisposition to develop this preference [28], might also have been acquired at some point throughout human evolution [12]: “It is possible that our brains have evolved to detect sharp features rapidly, perhaps using low-level features such as spatial frequencies” [12]. This fast detection and avoidance mechanism would have allowed individuals to detect and keep clear of sharp objects like thorns and pointed branches.

Some authors have proposed that this kind of preference was acquired after the human lineage separated from that of chimpanzees [29]. However, there is currently no evidence to support the notion that preference for curved contours actually is a derived human trait. In fact, there is reason to believe that the opposite might be the case. Other nonhuman primates exhibit diverse visual preferences, including the preference for regularity and symmetry [30], certain colors and brightness levels [31], and particular kinds of social information [32,33]. This is especially relevant when considering snakes, a well-researched threat to primates with a conspicuously curved contour [34]. The need to quickly detect such a threat might have helped to shape primate visual system [35], while it has been recently claimed that the rapid detection of snakes might be driven by key curved features of its body [36]. Thus, it is conceivable that the human preference for curved over sharp-angled contours evolved from preexisting cognitive and neural mechanisms related to visual preference present in our primate ancestors. If this were the case, visual preference for curved contours might be shared, at least to a certain degree, with other extant primate species.

The main aim of the present study was to test this possibility by comparing, under similar testing conditions, humans and nonhuman great apes’ (henceforth apes) preference for curved and sharp-angled contours. In the absence of an existing paradigm that allowed us to directly compare human and great ape visual preference, we designed an *ad hoc* experimental paradigm to test preference for contour curvature in humans (Experiments 1 & 2) and apes (Experiments 3 & 4). Our procedure was based on the approach-avoidance framework whose naturalistic essence and focus on primal interactions between organism and environment allowed us to minimize interpretative problems [37].

Participants were required to choose between two visual stimuli presented on a computer screen. The stimuli depicted objects that differed either only in their contour (thus, two versions of the same object, one with curved contour, the other with a sharp-angled contour) or in their content (both alternatives had the same sort of contour but depicted different objects). To simulate the effect of approaching the object, immediately after the choice had been made, the selected image was enlarged on the computer screen. Previous studies have established that two-alternative forced-choice paradigms are useful for detecting spontaneous preferences in primates [30,38,39]. Moreover, since humans and other primates share the same basic visual system and low-level visual processing [40], this paradigm was deemed particularly suitable to detect preferences for curved over sharp-angled contours.

Experiment 1 attempted to replicate previous results [11] using a 2-alternative forced choice (2AFC) with an 80-ms stimuli presentation time in humans. Based on the literature surveyed above, we predicted that humans would show a preference for curved objects by selecting the curved object above chance levels (50%). Given that previous studies using long or unrestricted viewing times have produced inconsistent results (e.g. [14], [16]), Experiment 2 was designed to study humans’ preference for curvature under unrestricted time conditions, also using a 2AFC. We hypothesized that the aforementioned inconsistencies are a reflection of the attenuation of preference for curvature with longer presentations. Thus, we expected to find a reduction in participants’ choices of the curved items in comparison to Experiment 1. Experiments 3 and 4 tested preferences in chimpanzees and gorillas using a touch screen with an 84 ms and unrestricted time stimuli exposure, respectively. We also expected apes to show a preference for curved objects, given the aforementioned evidence for other shared visual preferences. Nevertheless, we anticipated that the effects of longer exposure times would be different, owing to the weaker relevance of the semantic content of the presented items for apes.

We also analyzed response latency and choice consistency to attain a better understanding of the sources underlying participants’ choices. In this context, choice consistency indicates the frequency with which participants select the same item in the pairs when presented in two different experimental blocks. Thus, whereas the preference value reflects the extent to which participants’ choice is driven by contour, the consistency value reflects the extent to which this choice is linked to specific object pairs.

## Materials and Methods

### Experiment 1

Twenty psychology students from the University of the Balearic Islands (18 females, age *M* = 20.75, *SD* = 4.60, all adults) volunteered to take part. All of them were unaware of the goals of the experiment and had normal or corrected-to-normal vision. Experiments 1 and 2, and their consent procedure were approved by the Ethical Committee of the Comunitat Autònoma de les Illes Balears (Spain). Participants provided written informed consent to take part in the experiments.

**Fig 1:**
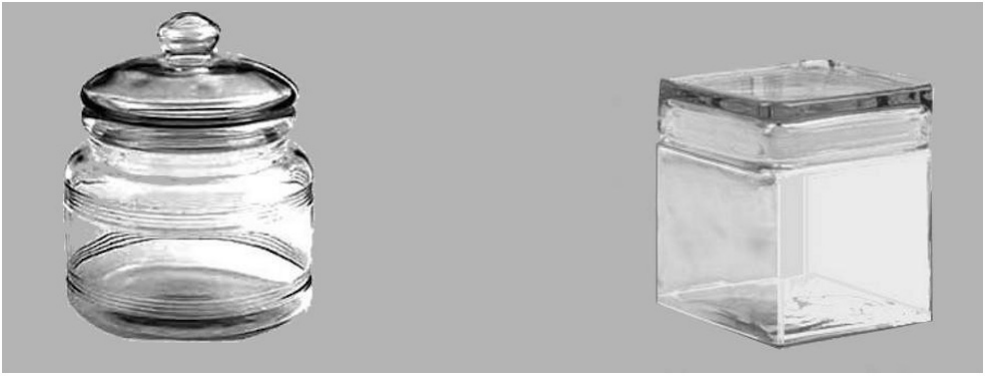
Example of contour pair. Same semantic meaning and different contour. From Bar & Neta (2006), used with their permission.

**Fig 2:**
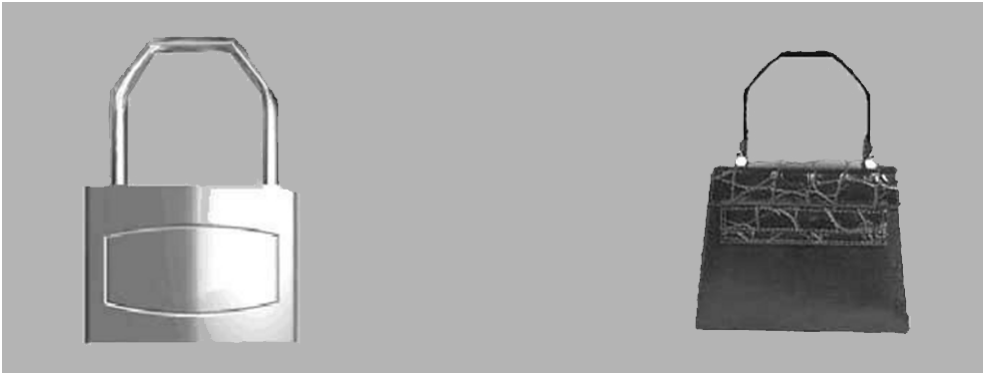
Example of content pair. Same kind of contour and different semantic meaning. From Bar & Neta (2006), used with their permission.

One hundred and sixty gray-scale photographs of real objects, a subset of those used in previous studies [11,12], were selected as stimuli. Each image had a resolution of 340 x 340 pixels so, when being shown on a 19-inch screen at 1440 × 900px (89.37 PPI), its real size was of 9.66 × 9.66 cm. The images were paired in order to create two sorts of pairings. A set of 36 contour pairs was created, each consisting of two versions of the same object that differed only in the curvature of its contour (one of the alternatives was curved, the other sharp-angled) (Fig 1). Additionally, 36 content pairs were created, consisting of different objects with the same sort of contour (curved or sharp-angled), thus differing in their semantic meaning (Fig 2).

These 72 pairs of images were distributed into two equivalent blocks. The two blocks were identical, except that, for each, pair the alternatives appeared on the opposite side of the screen. The order of both blocks was randomized, as was the order of the 72 trials in each block. This design enabled measuring lateralization effects and identifying other possible sources for preference, while at the same time increasing the number of trials. Eight additional training trials, comprising neutral stimuli that portrayed both curved and sharp-angled features, were added at the beginning of each session, so participants could become familiar with the procedure before the actual test began.

Each participant undertook only one session, in order to avoid undesired familiarity effects. They sat 50 to 60 cm from the screen in an isolated room, and were shown a total of 152 pairs of stimuli, consisting of the aforementioned 8 training pairs, and 36 contour pairs and 36 content pairs in each of the two blocks. Participants were instructed to select one of the images shown in a 2-alternative forced choice task by means of pressing a keyboard arrow that indicated the position of the selected image. These instructions were given avoiding the use of words in the semantic fields of *liking*, *preferring* or *wanting*, so participants were not led in a particular direction, and to facilitate inter species comparisons.

**Fig 3:**
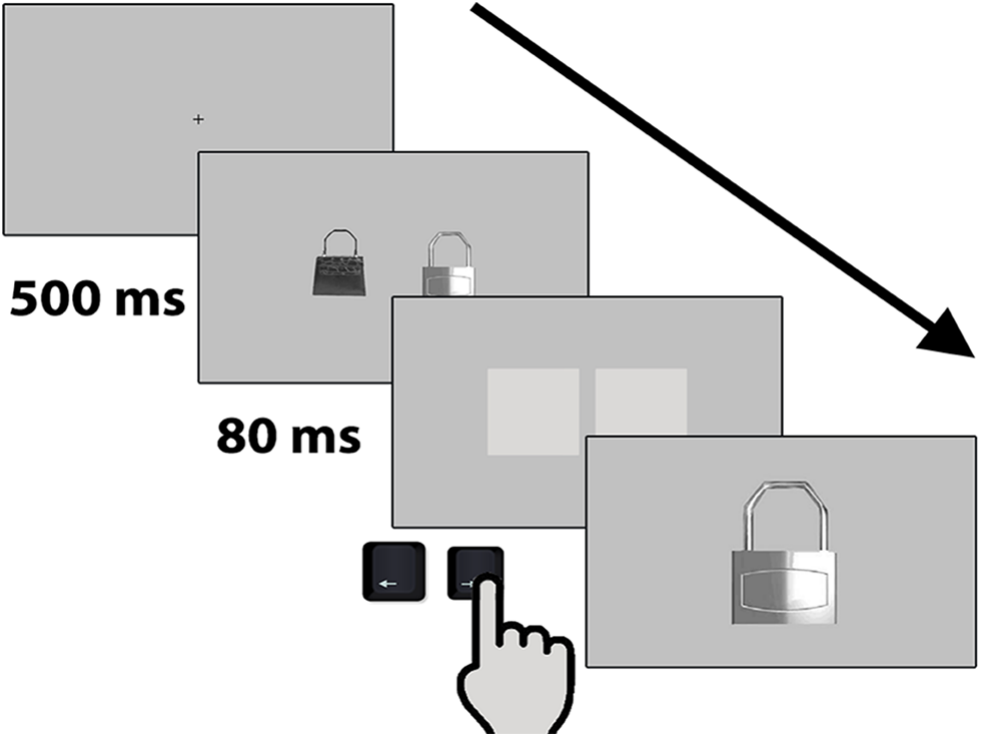
Trial sequence of Experiment 1. A fixation cross shown for 500 ms, followed by a pair of stimuli for 80 ms, immediately replaced by a pair of grey squares, and the chosen image was shown once again, centered, and enlarged.

A trial consisted of a fixation cross, shown for 500 ms, followed by a pair of stimuli displayed for 80 ms (Fig 3). This pair was then immediately replaced by a pair of grey squares, which minimized possible after-effects and served as a non-verbal cue signaling participants to make a choice. Once one of the options had been selected, the chosen image was shown once again for 1 second, centered, at twice its original size. This manipulation was aimed at 1) simulating the act of approaching the preferred image (by enlarging it) and 2) minimizing the task’s verbal requirements, thus enabling participants from different cultures and species to perform it [37].

Data were recorded by the computer and we measured three dependent variables: 1) choice preference, defined as the percentage of trials in which participants selected the curved contour alternative, 2) choice consistency, defined as the percentage of trials in which participants selected the same stimulus in the two experimental blocks and 3) latency, defined as the elapsed time between stimulus presentation and choice. Data were normally distributed and we used 2-tailed parametric statistical tests.

### Experiment 2

Twenty-nine students from the University of the Balearic Islands (24 women, age *M* = 19.86 *SD* = 1.78, all adults) took part in a 2 alternative forced choice task similar to that described in Experiment 1. The only difference was that the stimuli remained on the screen until the participant made a choice. All participants were unaware of the goals of the experiment and had normal or corrected to normal vision. Data analysis was the same as in Experiment 1.

### Experiment 3

Seven adolescent and adult chimpanzees (*Pan troglodytes;* 3 females) and two adult female western lowland gorillas (*Gorilla gorilla gorilla*) housed at the Wolfgang Köhler Primate Research Center (WKPRC) (age *M* = 14.22, *SD* = 5.21, Range: 8–20 years) took part in this experiment. One additional chimpanzee was removed from the study due to low performance during the training condition. In accordance with the recommendations of the Weatherall report “the use of non-human primates in research” groups of apes were housed in semi-natural indoor and outdoor enclosures with regular feedings, daily enrichment and water ad lib. Apes at WKPRC live in groups housed in separated outdoor (1400–4000 m^2^) and indoor enclosures (175–430 m^2^) that contain climbing structures, such as ropes and platforms; natural features, such as vegetation, trees and streams; and a variety of permanent enrichment devices. They spend the night in a series of interconnected sleeping rooms (32–47 m^2^), and receive regular feedings through the day consisting of a variety of fruits, vegetables and cereals. They are further provided with different kinds of enrichment devices once every day, with at least one item per individual (for more information, see http://wkprc.eva.mpg.de/english/files/enrichment.htm). Apes were not forced to participate and could choose to stop participating at any time during the study; they were never deprived of food or water. They were rewarded with highly valued food items, such as bananas, apples and grapes. Research was conducted in the observation rooms.

No medical, toxicological or neurobiological research of any kind is conducted at the WKPRC. Research was non-invasive and strictly adhered to Germany’s legal requirements. The study was ethically approved by an internal committee at the Max Planck Institute for Evolutionary Anthropology, which serves the same function as an Institutional Animal Care and Use Committee. Animal husbandry and research complied with the “EAZA Minimum Standards for the Accommodation and Care of Animals in Zoos and Aquaria”, the “WAZA Ethical Guidelines for the Conduct of Research on Animals by Zoos and Aquariums” and the “Guidelines for the Treatment of Animals in Behavioral Research and Teaching” of the Association for the Study of Animal Behavior (ASAB).

We used the same stimuli as in Experiment 1. Thus, humans and apes were presented with exactly the same choices. Like humans, the apes were required to perform a two-alternative forced choice task in which they had to choose between the same pairs of images previously described. But, in order to adjust to the particularities of working with non-human primates, several modifications were introduced, most notably the inclusion of an infrared touchscreen via which the apes made their selection. This screen was calibrated so its coordinates matched that of a 19-inch computer monitor placed right behind it, in which the black and white stimuli were presented at a resolution of 1280 x 1024 (86.27 PPI), achieving a real size of 10.01 x 10.01 cm per image. All participants were familiar with this setup as they had used it in other studies.

The apes could move freely in the observation room, so the fixation cross was replaced by an initialization cross between trials. This cross had to be touched each time in order for the stimuli to be displayed, thus helping direct the participant’s attention to the screen where the stimuli where shown. To further engage them in the task, the apes were rewarded in a quasi-random manner on 50% of the trials, such that they were not rewarded in consecutive trials and they did not go without a reward in more than two consecutive trials. To be consistent with previous training procedures, rewards were accompanied by a beeping sound associated with a correct response. Furthermore, to avoid any possible bias derived from an association between rewards and chosen images, the screen remained blank for 500 ms. after the enlarged selected image was shown. Following this, the beeping sound indicated whether the reward would or would not be delivered. Rewards were usually pieces of apple, although some pieces of grape and banana were also given. Finally, due to differences in the refresh rate of the computer screens used to present the stimuli to humans and apes, the latter viewed the images for 84 ms, compared to 80 ms for the humans in Experiment 1. Both presentation durations are consistent with previous literature [11,14].

Trials began with the initiation cross displayed on the computer screen. Once the ape touched it, a pair of images was shown for 84 ms and then replaced by two gray squares. Upon touching of one of these squares, the selected image reappeared, enlarged. After 1 sec of enlarged presentation, the computer screen remained blank for 500 ms, after which a beeping sound was presented depending on whether or not a reward was given.

In order to ensure that the apes could perform the task, they had to pass two training sessions on different days before proceeding to the experimental condition. These sessions differed only in that the stimuli presented were a subset of those used previously as control stimuli [11], comprising objects with “a roughly equal mixture of curved and sharp-angled features” [11]. The criterion for a successful training session was selection of a stimulus on every trial. Nine participants met criterion after two sessions; a tenth failed. Each ape received 5 identical experimental sessions on different days. Data analysis was the same as in Experiment 1.

### Experiment 4

This experiment was identical to Experiment 3, except that stimuli were presented until participants made a choice. As in Experiment 3, apes chose by touching one of the squares corresponding to the presented objects. As the same 9 apes that participated in Experiment 3 were tested, no additional training was necessary. Each ape again received 5 identical sessions across different days, and data analysis was the same as in previous experiments.

## Results

### Experiment 1

#### Preference

**Fig 4:**
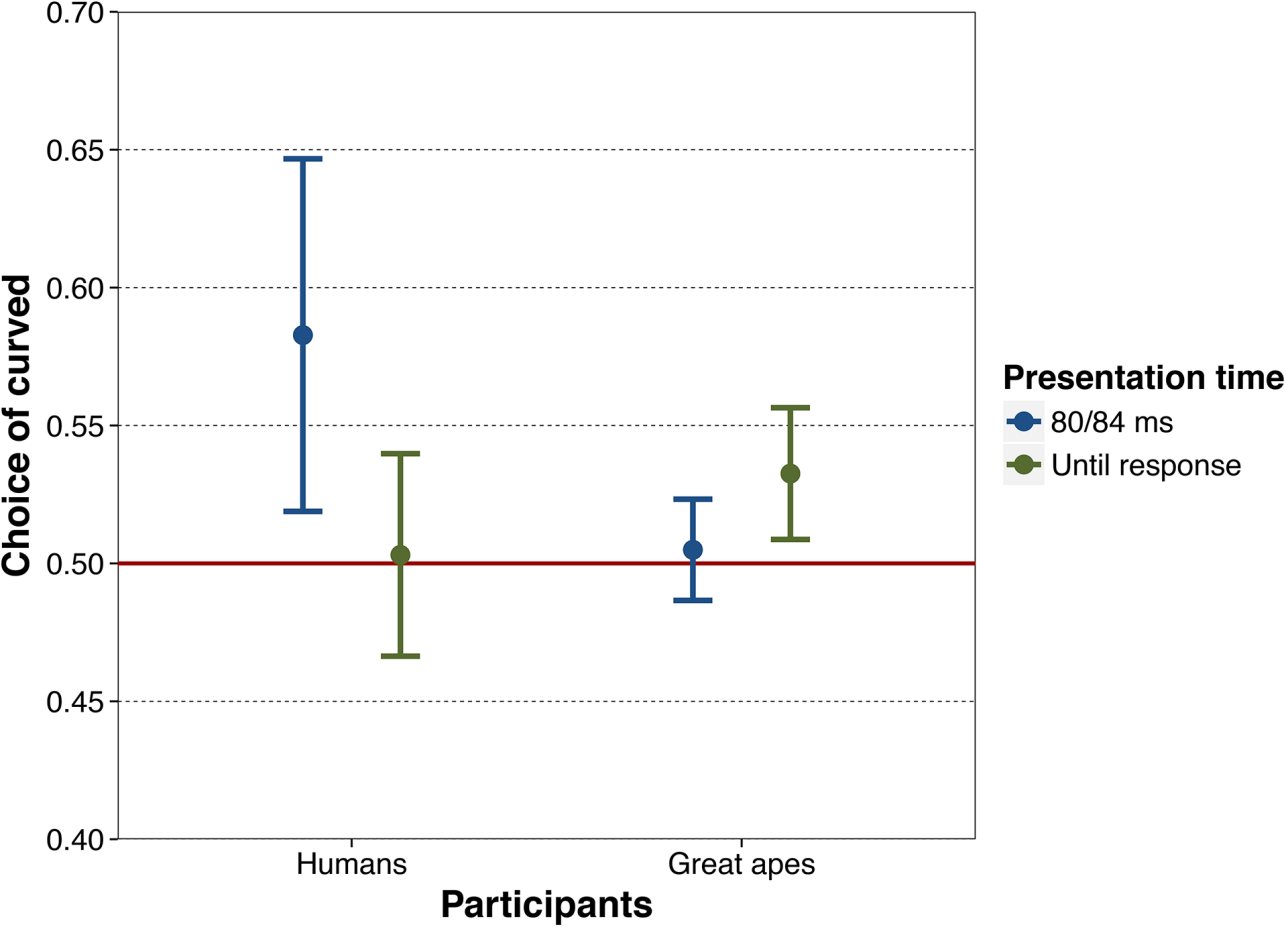
Proportion of curved stimulus choices by humans and great apes. From left to right, 95% confidence interval of the proportion of curved choices by humans when stimuli pairs were presented for 80 ms (Experiment 1) and until response (Experiment 2), and by great apes when stimuli pairs were presented for 84 ms (Experiment 3) and until response (Experiment 4). The red line at value .50 indicates chance-level choice.

As hypothesized, participants chose the curved alternatives in the pairs significantly above chance level [*t*(19) = 2.69, *p* = .007, *d =* .60] (Fig 4). We also performed an item-by-item analysis based on the number of participants that chose the curved item in every pair. Thus, this analysis was carried out on the 40 (20 participants x 2 blocks) trials for each pair. As expected, objects with curved contours were chosen above chance level [*M* = 58.25; *SD* = 8.2; 95% CI: 54.75–61.75; *t*(35) = 4.84, *p* < .001; *d* = .81]. Thus, the preference for objects with curved contours was observed across participants and across image pairs (with large effect sizes).

#### Consistency

**Fig 5:**
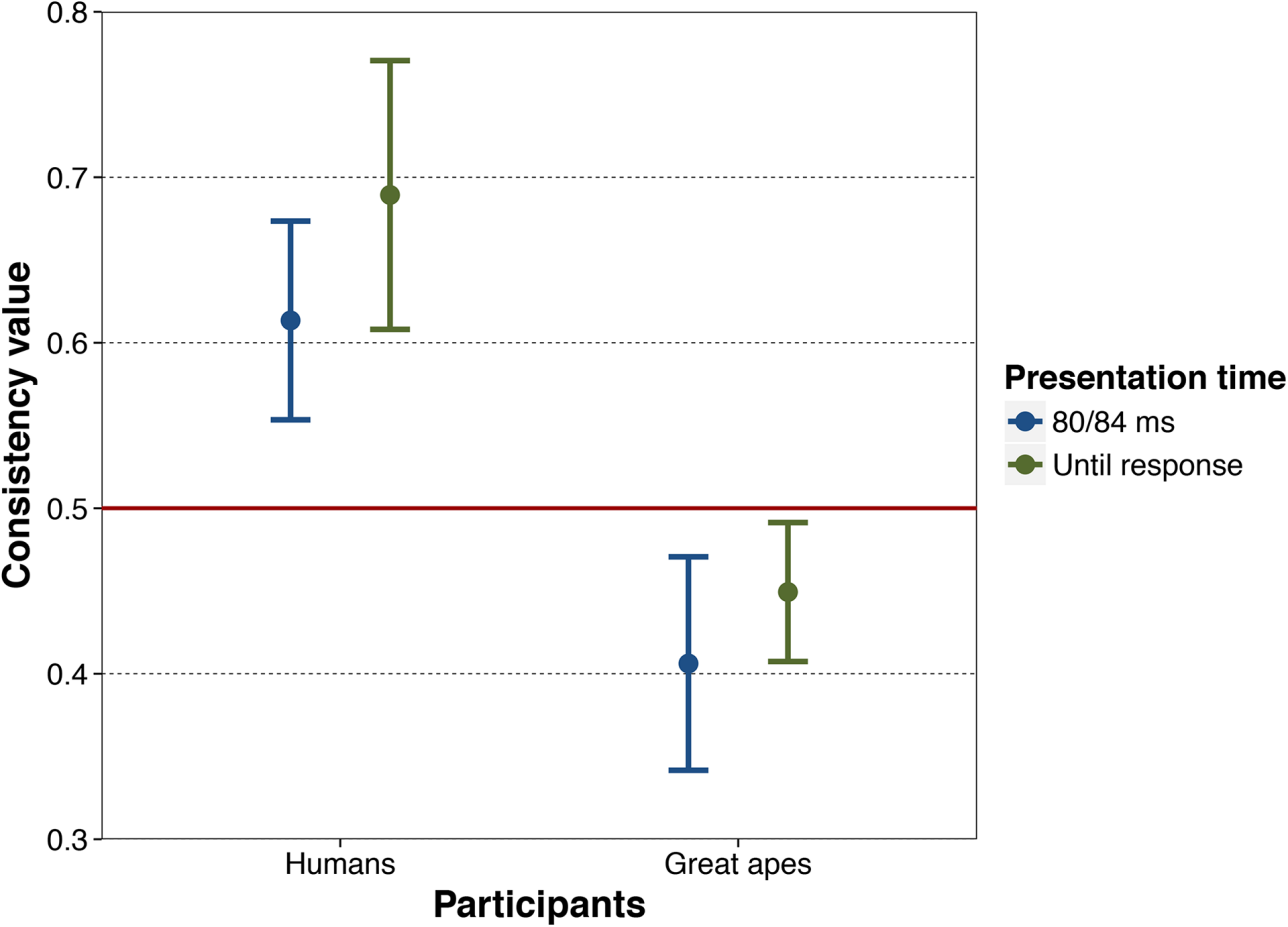
Consistency in the choice in humans and great apes. From left to right, 95% confidence interval of humans’ choice consistency when stimuli pairs were presented for 80 ms (Experiment 1) and until response (Experiment 2), and by great apes when stimuli pairs were presented for 84 ms (Experiment 3) and until response (Experiment 4). The red line at value .50 indicates chance-level consistency.

Choice consistency, defined as the percentage of pairs in which a participant chose the same image in the first and the second blocks, was above chance level [*t*(19) = 4.54, *p* < .001, *d* = 1.016] (Fig 5), with no difference between contour pairs [*M* = 61.7, *SD* = 13.17] and content pairs [*M* = 60.7, *SD* = 12.50], [*t*(19) = .33, *p* = .746, *d* = .08]. This indicates that participants preferentially chose the same item in the object pairs on both occasions.

#### Latency

We found no significant difference in average reaction time (RT) between the chosen curved alternatives [*M* = 625 ms, *SD* = 148 ms] and sharp-angled alternatives [*M* = 629 ms, *SD* = 173 ms], [*t*(19) = .21, *p* = .839, *d* = .025]. Similarly, there were no significant differences in RT between right [*M* = 627 ms, *SD* = 156 ms] and left responses [*M* = 625 ms, *SD* = 157 ms], [*t*(19) = .22, *p* = .828, *d* = .016] or between contour pairs [*M* = 628 ms, *SD* = 152 ms] and content pairs [*M* = 622 ms, *SD* = 160 ms], [*t*(19) = .46, *p* = .653, *d* = .038].

### Experiment 2

#### Preference

Data from two outlier participants were excluded from the analyses, given that their values exceeded the 1.5 × IQR (interquartile range) mark. Results for the remainder of participants show that they did not choose the curved alternatives above chance level under free viewing time conditions [*t*(26) = .173, *p* = .864, *d =* .03] (Fig 4). This was still the case when including the 2 outliers in the analysis.

#### Consistency

Preferences were highly consistent across blocks [*t*(26) = 6.5, *p* < .001, *d* = 1.25] (Fig 5), with choices of the content pairs being significantly more consistent than those of the contour pairs [*t*(26) = 3.29, *p* = .003, *d* = .39; content pairs: *M* = 76.5, *SD* = 17.6; contour pairs: *M* = 68.9, *SD* = 20.5]. Thus, participants chose the same item in image pairs on both presentations more often than not. Thus, the number of image pairs for which participants’ choice was the same in both blocks was greater when the pairs differed in content than when they differed in contour.

#### Latency

There were no significant differences in the average reaction time (RT) when choosing curved alternatives [*M* = 946 ms, *SD* = 388 ms] or sharp-angled alternatives [*M* = 926 ms, *SD* = 390 ms], [*t*(26) = 1.19, *p* = .244, *d* = .05], or between right [*M* = 904 ms, *SD* = 359 ms] and left [*M* = 918 ms, *SD* = 370 ms] responses [*t*(26) = .93, *p* = .36, *d* = .054]. In contrast, the average RT for contour pairs [*M* = 935 ms, *SD* = 385 ms] was significantly longer than for content pairs [*M* = 884 ms, *SD* = 345 ms], [*t*(26) = 2.73, *p* = .011, *d* = .14].

### Experiment 3

#### Preference

The percentage of items with curved contours chosen by each of the individual great apes after an 80 ms exposure is shown in the S2 File. Overall, apes did not preferentially select objects with curved contours above chance level [*t*(8) = .62, *p* = .276; *d =* .207] (Fig 4).

#### Consistency

Choice consistency was below chance level [*t*(8) = 3.45, *p* = .009, *d* = 1.15] (Fig 5) with no significant difference between contour and content pairs [*t*(8) = .39, *p* = .703, *d* = .081, contour pairs: *M* = 40.6, *SD* = 8.4, content pairs: *M* = 39.9, *SD* = 9.4]. Thus, for any object pair, the item selected in the first block did not necessarily correspond to the item selected in the second block, revealing that apes’ choices were largely unrelated to the identity of the objects in the pairs.

#### Latency

There were no significant difference in average RT between the curved alternatives [*M* = 673 ms, *SD* = 183 ms] and sharp-angled alternatives [*M* = 670 ms, *SD* = 167 ms], [*t*(8) = .09, *p* = .927, *d* = .018]. Similarly, there were no significant differences in RT between right [*M* = 764 ms, *SD* = 253 ms] and left choices [*M* = 694 ms, *SD* = 233 ms], [*t*(8) = .74, *p* = .482, *d* = .287] or between contour pairs [*M* = 672 ms, *SD* = 168 ms] and content pairs [*M* = 711 ms, *SD* = 185 ms], [*t*(8) = 1.85, *p* = .101, *d* = .224].

### Experiment 4

#### Preference

The apes chose the objects with curved contours significantly above chance level [*t*(8) = 3.15, *p* = .007; *d =* 1.05] (Fig 4). An item-by-item analysis based on 90 trials (9 participants x 2 blocks x 5 sessions) revealed that participants chose objects with curved contours above chance [*M* = 53.2; *SD* = 8.2; 95% CI: 50.4–56.0; *t*(35) = 2.36, *p* = .012, *d* = .393]. Therefore, preference for curved objects was observed both across participants (with a large effect size) and across image pairs (with a moderate effect size). An analysis of the course of apes’ choices over the 5 test sessions indicated that apes chose curved versions of the target pairs significantly above chance level even in the first session (they chose these alternatives on 57.1% of the trials; *t* = 2.208, *p* = .027, 95% CI: 50.8–63.1%), and that this pattern did not vary significantly across sessions (S2 File). Finally, a study of the effects of the random rewards showed that apes’ choices in the second block were unrelated to whether their choices in block 1 had been followed by a reward or not (S2 File).

#### Consistency

**Fig 6:**
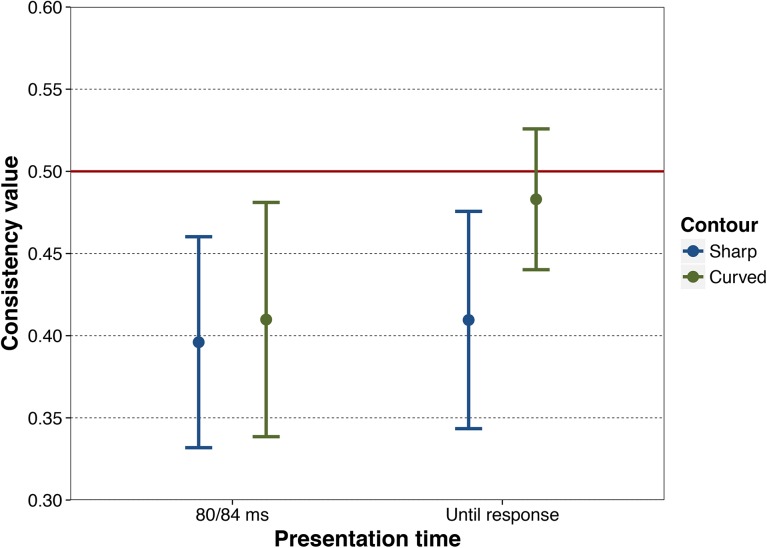
Consistency in the choice of curved and sharp-angled contours only in great apes. Consistency results from Experiments 3 and 4. From left to right, 95% confidence interval of great apes’ choice consistency when stimuli pairs were presented for 84 ms for sharp-angled and curved contours (Experiment 3), and when stimuli pairs were presented until response for sharp-angled and curved contours (Experiment 4). The red line at value .50 indicates chance-level consistency.

Consistency was significantly below chance [*t*(8) = 2.84, *p* = .022, *d* = 0.95] (Fig 5) with no difference between contour pairs [*M* = 44.9, *SD* = 5.46] and content pairs [*M* = 44.7, *SD* = 6.95], [*t*(9) = .12, *p* = .906, *d* = .04]. However, apes were significantly more consistent when in the first block they chose the curved compared to the sharp-angled alternative [*t*(8) = 2.51, *p* = .036, *d* = 1.04] (Fig 6). Thus, the apes were more likely to choose the same alternative if in the first block they had chosen the curved rather than the sharp-angled one.

#### Latency

There was no significant difference in RT between the curved alternatives [*M* = 849 ms, *SD* = 123 ms] and sharp-angled alternatives [*M* = 793 ms, *SD* = 203 ms], [*t*(8) = 1.16, *p* = .28, *d* = .34]. Similarly, there were no significant differences in RT between right [*M* = 830 ms, *SD* = 227 ms] and left responses [*M* = 848 ms, *SD* = 150 ms], [*t*(8) = .31, *p* = .767, *d* = .097] or between contour pairs [*M* = 827 ms, *SD* = 151 ms] and content pairs [*M* = 799 ms, *SD* = 192 ms], [*t*(8) = 2.08, *p* = .095, *d* = .369].

### Discussion and Conclusions

We found a double dissociation in choice preference and choice consistency between the human and ape groups tested in this study when confronted with pairs of objects that differed in the aspect of their contour. Regarding the choice patterns, both the human group and the ape group showed a preference for objects with curved (as opposed to sharp-angled) contours, albeit under different presentation conditions. In particular, the human group preferred objects with curved contours under brief presentation conditions (with a large effect size), but not under free viewing conditions. In contrast, the ape group showed the reverse pattern: they preferred objects with curved contours under free viewing conditions (with a moderate effect size), but not under brief presentation conditions. With regard to consistency, in each of their two experiments the human group showed choice consistency above chance levels, whereas the ape group showed choice consistency below chance levels. Participants in the human group tended to select in the same way when presented with the same stimuli pairs a second time, whereas those in the ape group did not. Next we discuss the implications of each finding in turn.

Human participants exposed to pairs of objects for 80 ms showed a preference for objects with curved contours over objects with sharp-angled contours. Our results are comparable to the values reported in previous studies [10–16]. This finding reinforces the notion that preference for curved contours is a robust finding, stable across different experimental paradigms. In contrast, when presented with the same stimuli under free viewing conditions, the human group showed no preference for curved contours. Interestingly, the only previous study failing to find a marked behavioral effect of curved contours [16] involved long presentations and used a behavioral approach-avoidance procedure similar to our own (Experiment 2). Although some other studies using long presentations reported an effect of curvature [10,13,15], participants were required to respond to questions about value judgments (attractiveness, pleasantness, liking, or beauty), rather than to produce an overt motor response.

The preference for curved contours with short but not long presentation times in the human group was reversed in the great ape group (Experiments 3 and 4). Unlike the human group, the great ape group showed no preference for objects with curved contours when presented for 80 ms (Experiment 3) but showed a preference under free viewing time conditions. It is unlikely that this disparity occurred because participants in the ape group were unable to perceive stimuli when presented for short durations. Research using visual masking paradigms—which measure visibility of a brief visual target followed by a mask—has shown that both humans and chimpanzees are able to perceive and respond to stimuli with 60 ms temporal asynchrony between target and mask [41]. Given that the presentation time in our experiments was slightly longer, the lack of preference for curved contours in the group of apes when stimuli were presented for short durations requires another explanation. One possibility is that ape participants were not looking at the screen continuously, therefore missing the briefly presented stimuli in some trials. Another possibility has to do with differences between humans and apes in processing visual objects. Humans show an advantage over chimpanzees in processing global features of objects [40, 42]. In our brief presentation task, thus, the global qualities of the presented objects—including contour, whether curved or sharp-angled—might have played a greater role in the decisions of participants in the human group than in the decisions of participants in the ape group. Further research is required to better understand how these factors might affect individuals’ responses.

The human and ape groups differed in their choice consistency. Even when contour did not influence human participants’ choice (under long stimuli presentations), their consistency scores were very high, even higher than when stimuli were presented for 80 ms. Their choices, thus, were not arbitrary, but tended to be systematic. Moreover, content pairs (depicting different objects with the same sort of contour) showed higher consistency than contour pairs (depicting the same object with different contours). This suggests that participants’ choices were driven mainly by content, meaning, or preferences for other visual features. Thus, under longer presentation times, the human group’s initial behavioral preference for curved contours, related to low-level features, may be superseded by more elaborate choices related to the content, semantic processing [14], or to other sources of visual preference.

In the ape group, choice consistency for both brief and free-viewing presentation modes was below 50%. It is conceivable that this result simply reflects the way the task was implemented. Apes were rewarded regardless of their response. Given that responses to either side of the touchscreen were followed by reinforcement that was response independent, they might have responded on the same side of the touchscreen for convenience. In other words, if one participant chose to respond mostly on one side and kept doing in the second block, were the alternatives in each pair were presented on opposite sides of the screen, the consistency necessarily dropped below 50%. This explanation is supported by the strong lateral bias, especially with short presentations, and the large variability among individuals.

One important goal of our study was to develop an experimental paradigm that could be administered to both humans and apes. This is important because the field of comparative cognition often relies on indirect comparisons across species, and requires more studies that test species on the same tasks [43]. Nevertheless, “there is no single method that can be applied without bias across taxa” [43], and although we have sought to standardize the essential features of our paradigm, some differences remain between the experiments carried out with the group of humans and the group of apes, something that is a common practice in comparative psychology [44, 45]. In our study, humans responded by using a keyboard and apes by using a touchscreen, humans performed the experiment in one session whereas apes received 5 sessions, and apes received random food rewards to maintain their motivation but humans did not. Note that we found that apes’ preferences were not affected by the reward regime (S2 File).

In sum, our results showed that the human and ape groups shared a preference for curved over sharp-angled contours. Future studies are required to replicate and extend these results to individuals belonging to different cultures and with different upbringing histories. Although our data cannot refute the possibility that such preferences evolved independently in humans and apes, it is possible that the human preference for curved objects and avoidance of sharp-angled ones evolved from visual preferences in the common ancestor of humans, chimpanzees, and gorillas. Throughout the evolution of the human lineage this visual preference for curved contours seems to have become stronger, perhaps due to the increasing relevance of global configuration processing, and susceptible to the influence of semantic information and preferences for other perceptual qualities.

## Supporting Information

S1 File The ARRIVE Checklist. Animal Research Guidelines: Reporting In Vivo Experiments.
(PDF)

S2 File Tables and other results. Individual results in apes, analysis whether apes choices were influenced by multiple test sessions and analysis of the influence of the rewards administered in block 1 on choices in block 2.
(PDF)

S3 File Data Supporting Information. Raw data from experiments 1, 2, 3, and 4.
(XLSX)
