# Supplementary material for: Common Visual Preference for Curved Contours in Humans and Great Apes
Source: PLoS One. 2015 Nov 11;10(11):e0141106. doi: 10.1371/journal.pone.0141106 (PMC4641621; doi:10.1371/journal.pone.0141106)
Supplement: S2 File — Individual results in apes, analysis whether apes choices were influenced by multiple test sessions and analysis of the influence of the rewards administered in block 1 on choices in block 2. (PDF) [file pone.0141106.s002.pdf]

Tables

Table 1. Great apes' choices when stimuli pairs were presented for 84 ms.

	Name	Choice of curved	Choice of right	Consistency
Chimpanzees	Trudi	46.4 n.s.	71.3	41.4
	Jahaga	48.1 n.s.	69.4	41.1
	Fifi	50.3 n.s.	10.4	19.7
	Alex	51.4 n.s.	30.7	41.4
	Lome	49.2 n.s.	72.8	35.6
	Kofi	51.9 n.s.	42.1	46.1
	Lobo	53.1 n.s.	48.2	45.3
Gorillas	Kibara	53.9 *	50.4	47.5
	Viringika	50.3 n.s.	36.2	44.2
	Total	50.5	47.9	40.2

Percentages of choices for the curved object, for the object on the right, and the consistency between both blocks.

Binomial test, n.s.: non significant, * < .05.

Table 2. Great apes' choices when stimuli pairs were presented until response.

	Name	Choice of curved	Choice of right	Consistency
Chimpanzees	Trudi	49.7 n.s.	35.1	48.3
	Jahaga	52.2 n.s.	68.6	42.2
	Fifi	51.9 n.s.	66.8	46.1
	Alex	54.2 *	55.1	37.2
	Lome	50.8 n.s.	19.9	37.2
	Kofi	55.4 *	35.3	48.9
	Lobo	56.1 *	56.2	51.1
Gorillas	Kibara	58.9 *	29.2	42.2
	Viringika	50.0 n.s.	39.9	51.1
	Total	53.3	45.1	44.9

Percentage of choices for the curved object, for the object on the right, and the consistency between both blocks.

Binomial test, n.s.: non significant, * < .05.

EXPERIMENT 4: Linear mixed effects model to analyze whether apes' choice was influenced by multiple test sessions.

- Were apes' choices influenced by the fact that they were required to perform 5 test sessions? In order to answer this question we used linear mixed effects modeling to determine whether apes' choice of curved versions in test session 1 differed from chance levels, and whether this pattern changed over the course of the subsequent 4 test sessions.

Analysis design:

- The factor Session was dummy coded, and Session 1 was used as reference level
- The factor Session was included to test whether this prediction varied from session 1 to each of the other sessions.
- To tell us about the variability among apes and among stimuli, the model took into account simultaneous within- and between- subject effects.

Results:

- In session 1 (reference level) apes chose the curved-contour version of the stimuli significantly above chance, specifically on 57.1% of the trials (t = 2.208, p = .027, 95% CI: 50.8 - 63.1%)
- Differences between the proportion of apes' curved-contour choices did not differ significantly among the 5 sessions (all ps > .13)
- Likelihood ratio tests revealed that these results were highly consistent among the apes ($\chi^2(14) = 9.636$; p = .788).

EXPERIMENT 4: Linear mixed effects model to analyze whether the random rewarded choice regime in the Block 1 influenced apes' their choice in the Block 2.

- Did rewards administered to apes during Block 1 influence their choices during Block 2? In order to answer this question we tested whether apes' choices in Block 2 could be predicted by the interaction between choices in Block 1 and whether these choices had been followed by a reward.

Analysis design:

- If the rewards administered in Block 1 impacted choices in Block 2 we would expect a significant interaction between the response in Block 1 and Rewards in Block 1 in predicting choice in Block 2.
- The factor Session was included to test whether this prediction varied from session to session.
- To tell us about the variability among apes and among stimuli in the predictive strength, the model took into account simultaneous within- and between- subject effects.

Results:

- The three-way interaction (Choice in Block 1 × Reward × Session) was not significant (for all contrasts, *p*s > .37), and neither was the two-way interaction Choice in Block 1 × Reward (β = -0.09694; z = -0.472; p = .637). Thus, apes'

choices in Block 2 for any given pair were unrelated to whether their choices for that pair in Block 1 had been followed by a reward or not, and this did not change from session to session.

- Likelihood ratio tests revealed that this result was highly consistent among the apes ($\chi^2(9) = 7.100$; $p = .627$).
